# Determinants of Diabetes-Related Quality of Life in Saudi Arabia: A Nationwide Survey-Based Assessment of Demographic, Health, and Complication-Related Influences

**DOI:** 10.3390/medicina61091583

**Published:** 2025-09-01

**Authors:** Ebtihag O. Alenzi, Alya AlZabin, Ebtesam Almajed, Norah Alqntash

**Affiliations:** 1 Family and Community Medicine Department, College of Medicine, Princess Nourah bint Abdulrahman University, P.O. Box 84428, Riyadh 11671, Saudi Arabia; 2 College of Medicine, Princess Nourah bint Abdulrahman University, P.O. Box 84428, Riyadh 11671, Saudi Arabia; 439001489@pnu.edu.sa (A.A.); 439003552@pnu.edu.sa (E.A.); 439001062@pnu.edu.sa (N.A.)

**Keywords:** diabetes mellitus, quality of life, comorbidity, complications, health behavior

## Abstract

*Background and Objectives*: Diabetes affects quality of life (QoL) in physical, psychological, and social aspects. With high prevalence rates in Saudi Arabia, this study aimed to assess QoL in adults with diabetes across different regions and identify associated factors, addressing research gaps on complications and demographic influences. *Materials and Methods*: This cross-sectional study recruited adults diagnosed with diabetes from all regions of Saudi Arabia through phone interviews and the distribution of self-administered questionnaires via social media platforms. Data on demographics, health behaviors, diabetes-related complications, comorbidities, and diabetes management were collected. QoL was assessed using the Diabetic Quality of Life (DQoL) tool. Statistical analysis included descriptive statistics and multivariate regression, with significance set at *p* < 0.05. *Results*: Among the 527 individuals with diabetes, 57.7% were married, 56.1% were female, 93.7% lived in urban areas, and 37.2% had at least a bachelor’s degree. Common comorbidities included hypertension (29.2%) and hypercholesterolemia (22.8%). Physical activity and healthy weight were linked to improved DQoL. Diabetic complications, such as neuropathy (6.6%) and diabetic foot (4.9%), were significantly associated with lower DQoL scores. Depression was reported in 4.7% of participants and was the strongest predictor of poor QoL (β = −2.01, *p* < 0.001). Furthermore, individuals who exercised less than five times per week had significantly lower QoL scores (beta = −1.83; 95% CI = −2.56 to −1.10; *p*-value < 0.001). *Conclusions*: The study highlights the significant impact of education, health behaviors, diabetes complications, and comorbid depression on QoL. Comprehensive diabetes care that addresses both physical and psychological factors is essential for achieving improved outcomes.

## 1. Introduction

Diabetes is a group of chronic metabolic disorders marked by persistent hyperglycemia due to defects in insulin secretion, action, or both. Prolonged hyperglycemia can lead to damage in various organs, including the eyes, kidneys, nerves, heart, and blood vessels [1]. It encompasses several types, including Type 1 Diabetes Mellitus (T1DM), Type 2 Diabetes Mellitus (T2DM), and gestational diabetes, with T2DM being the most prevalent form among adults. Globally, DM imposes a substantial burden on healthcare systems, contributing significantly to morbidity, mortality, and increasing health expenditures [2]. Approximately 529 million individuals had DM worldwide in 2021, with North Africa and the Middle East having the highest age-standardized rates [3]. In Saudi Arabia, the prevalence of diabetes was about 16.4% among the population, and this prevalence varied considerably across different regions [4].

This high prevalence significantly affects individuals’ physical, psychological, and social functioning, diminishing their quality of life (QoL) [5]. Common impairments include pain, discomfort, and mobility limitations, while issues with usual activities are less frequently reported [6]. Globally, instruments, such as the diabetes-specific quality of life scale (DSQOLS) and the diabetes treatment satisfaction questionnaire (DTSQ), have shown that poor glycemic control is associated with lower QoL [7]. Numerous studies have linked reduced QoL to age, diabetes duration, insulin use, obesity, and DM-related complications [6,8,9]. Among diabetes-related complications, neuropathy, retinopathy, nephropathy, and diabetic foot ulcers are prominent contributors to reduced quality of life. Notably, painful diabetic peripheral neuropathy has a substantial impact; a study from Saudi Arabia reported a prevalence of 33% and found significantly lower physical and mental QoL scores among those affected [10].

Lifestyle interventions, especially physical activity, are vital in diabetes management and closely linked to improved QoL. Interventional studies have shown that structured exercise enhances metabolic control, psychological well-being, and physical function. For example, Fanelli et al. and Schranz et al. reported significant improvements in metabolic outcomes, strength, and self-concept through supervised exercise in adolescents with obesity [11,12]. Despite this, structured physical activity programs remain limited among patients with diabetes. A cross-sectional study found that 26.3% of adults with type 2 diabetes met recommended activity levels, referring to cultural norms, and insufficient provider guidance as key barriers [13]. Structured primary care models and lifestyle-focused case management approaches are increasingly recognized as effective in supporting diabetes self-management and improving QoL [14].

In Saudi Arabia, diabetes has been shown to adversely affect health-related quality of life (HRQoL) and mental health. A study in the Southern Province, using the WHOQoL-BREF, found that lower scores were associated with older age, female gender, longer disease duration, comorbidities, and lower educational levels [15]. At the same time, better outcomes were associated with a healthy diet, regular physical activity, and appropriate therapy [15]. Another study, using the EuroQol-5 dimensions-three levels (EQ-5D-3L) and EuroQol-visual analogue scale (EQ-VAS) tools, found that younger, male, married, and employed individuals with higher socioeconomic status reported better HRQoL compared to their counterparts [16].

Although interest in QoL among patients with diabetes has grown, many studies have lacked representative samples, standardized tools, and detailed assessment of complication-specific impacts. Therefore, research using a validated, disease-specific instrument in a larger, more diverse population is needed. Identifying predictors of QoL is essential to inform targeted prevention and management strategies. The primary aim of this study was to estimate the QoL of adults with diabetes in Saudi Arabia using a validated and reliable instrument. The secondary aim was to examine the factors, including complication-specific variables, which may influence QoL across different regions.

## 2. Method

### 2.1. Study Design

This cross-sectional study adhered to the STROBE (Strengthening the Reporting of Observational Studies in Epidemiology) guidelines for observational research [17], using a convenient sample of participants from all regions of Saudi Arabia. A completed STROBE checklist is provided in the Supplementary Material.

### 2.2. Sample Size

The sample size was calculated based on an estimated diabetes prevalence in Saudi Arabia [18], with a 95% confidence level and a 5% margin of error. A moderate effect size was assumed, resulting in a minimum required sample size of 227 participants. To accommodate potential data loss due to incomplete responses or exclusion criteria, a target sample size of at least 300 participants was established. Ultimately, a total of 527 participants were included in the final analysis. To confirm the adequacy of this sample, a post-hoc power analysis was conducted using G*Power version 3.1.9.7 (Institute for Experimental Psychology, Heinrich Heine University Düsseldorf, Düsseldorf, Germany). Assuming a moderate effect size (f^2^ = 0.15), an α level of 0.05, and 44 predictors, the achieved power with a sample size of 527 was 0.9997, indicating excellent power to detect statistically significant associations.

### 2.3. Procedures

After obtaining Institutional Review Board (IRB) approval, data collectors were recruited to distribute the survey in all administrative regions of Saudi Arabia between December 2023 and March 2024. Inclusion criteria were individuals who lived in Saudi Arabia, agreed to participate in the study, and had been diagnosed with DM. Exclusion criteria included individuals under 18 years of age and pregnant women. Participants completed the survey through interviews or by self-administered questionnaires distributed through several social media platforms, specifically X, WhatsApp, and Telegram. For interviews, data collectors conducted phone-based interviews using the same questionnaire format as the online version. Respondents in the interviews answered questions verbally, and their responses were recorded directly by the data collectors onto the digital form. The participants with diabetes who agreed to participate were included. The informed consent form was obtained from participants to enable them to complete the questionnaire. Anonymity was preserved by avoiding the collection of any personal identifiers. Each response was assigned a randomized, de-identified code, and all data were stored securely, ensuring that they were not linked to the respondent’s identity. Participants may choose not to answer some or all the questions.

### 2.4. Measures

The independent variables assessed in this study encompassed demographic and socioeconomic characteristics, healthcare access, health behaviors, diabetes-related factors, comorbidities, and diabetes management strategies. Demographic and socioeconomic variables included age, gender, marital status, educational level, occupation, region of residence, monthly family income, number of household members, and nationality.

Healthcare access variables comprised medical insurance status, primary healthcare service utilized, out-of-pocket expenditures for diabetes care per month, number of hospitalizations due to diabetes or its complications in the last 12 months, and hospitalizations due to other conditions in the last 12 months.

Health behavior variables included lifestyle-related factors such as diet, physical activity, smoking status, and self-reported weight (in kilograms) and height (in centimeters). Body Mass Index (BMI) was calculated and categorized into four groups: underweight, normal weight, overweight, and obese.

Diabetes-related variables included duration of diabetes, family history of the condition, and diabetes-related complications such as retinopathy, nephropathy, diabetic foot, and diabetic coma.

Comorbidities were categorized into cardiovascular, metabolic, psychiatric, musculoskeletal, respiratory, and other chronic conditions as reported by participants. Diabetes management was evaluated based on the use of metformin, other oral antidiabetic agents, insulin therapy, and complementary medicine.

The primary outcome of interest was the QoL that was assessed using the validated Diabetic Quality of Life (DQoL) [19]. This disease-specific tool was selected over generic instruments (e.g., SF-36, EQ-5D-5L) due to its higher sensitivity in capturing the multifaceted impact of diabetes on patients’ daily lives, including treatment burden and psychosocial dimensions. The DQoL consists of 46 items across three domains: Satisfaction (15 items), impact (20 items), and Worry (11 items), which is further divided into social/vocational and diabetes-specific concerns. Items are rated on a 5-point Likert scale, where items in the Impact (except item #5) and Worry domains were reverse-coded to make the results easier to interpret, with higher scores indicating better QoL. The total score is calculated by summing responses of all items per participant and dividing by the number of items to generate a mean DQoL score out of five.

### 2.5. Statistical Analysis

Means with standard deviation (SD) were reported for continuous variables, and frequencies with percentages were reported for categorical data. The bivariate associations of sample-related factors with the sample’s total DQoL score were examined using Student’s independent *t*-tests and one-way analysis of variance (ANOVA). The adjusted associations of the patients’ DQoL with demographic and socioeconomic factors, access to healthcare, health behaviors, diabetes-related factors, comorbidities, and diabetes management were examined using multivariate ordinary least squares regression. Test results of *p*-value < 0.05 were considered significant. The statistical analysis was performed using the Statistical Package for Social Sciences SPSS Version 25 (SPSS, Inc., Chicago, IL, USA).

## 3. Results

Of the 882 individuals who received the survey, 14 declined to participate, and 341 were excluded for not reporting a diagnosis of diabetes. Thus, 527 individuals with confirmed diabetes were included in the analysis, yielding a final response rate of 97.3%. The high response rate may be attributed to the personalized distribution methods, the nationwide coverage of data collectors across all regions, and the clear, concise survey format. No monetary incentives were offered, but the data collectors were trained to encourage completion by emphasizing the importance of the research.

### 3.1. Sample’s Characteristics

The sociodemographic characteristics of the sample are demonstrated in Table 1. The study sample had a broad age distribution, with the majority falling between 40 and 60 years old, comprising 56.1% females and 43.1% males. Most participants were married (57.7%), and the majority had at least a bachelor’s degree. Regarding occupation level, 34.7% were working, while 25.8% were housewives, and a small proportion were retired (14.0%) or students (13.1%). Most participants reported a monthly family income between 5000 and 10,000 SAR (21.6%). Geographically, 40.4% of the population resided in the central region of Saudi Arabia, and a vast majority (93.7%) lived in urban areas. Most participants were Saudi (88.8%), while 11.2% were non-Saudi. The most common household size ranged between four and six family members.

Table 2 displays the healthcare access and healthcare utilization of the included participants. Over half of the participants (54.1%) were non-insured and relied on public healthcare facilities, while only one-third (33.6%) had medical insurance. The most accessed settings for diabetes care were public hospitals (53.1%). Outpatient clinic services were the most prevalent type of service used (79.9%), followed by limited use of emergency services (11.6%) and pharmacy services (8.5%). In terms of financial burden, more than half (51.0%) reported no out-of-pocket expenditures, while 26.5% incurred moderate to high monthly costs related to diabetes. Notably, 70.6% of the participants had no history of hospitalization due to diabetes or its complications, and 64.1% had not been hospitalized for other health conditions.

Table 3 summarizes the health behaviors, diabetes-related factors, and comorbidities of the participants included in this study. A significant proportion of participants were either overweight (31.9%) or obese (38.0%), and over half (53.9%) reported not following a healthy diet. Physical inactivity was prevalent, with 35.7% of participants reporting no regular exercise. While most participants were non-smokers (76.9%), 14.4% were current smokers. Regarding diabetes-related characteristics, the most common disease duration was less than 5 years (30.2%), and a substantial proportion had a first-degree family history of diabetes (43.1%). The prevalence of diabetes-related complications included retinopathy (12.3%), nephropathy (11.4%), neuropathy (6.6%), diabetic foot (4.9%), and diabetic coma (3.8%). Additionally, 59.4% reported having at least one comorbid condition. In terms of diabetes management, metformin was the most used medication (64.5%), followed by insulin injections (52.6%), while the use of complementary medicine was relatively low (10.1%).

**Figure 1:**
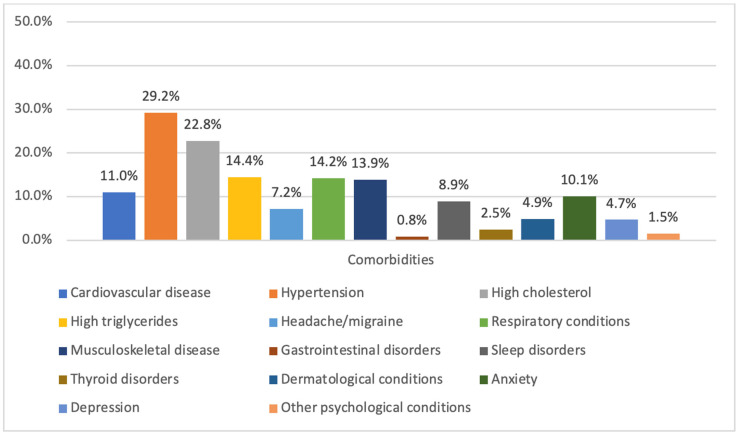
Percentage of comorbidities among the included participants.

Figure 1 illustrates the distribution of self-reported comorbidities among participants with diabetes included in the study. The most prevalent comorbid condition was hypertension (29.2%), followed by high cholesterol (22.8%), high triglycerides (14.4%), and respiratory conditions (14.2%). Musculoskeletal disease (13.9%) and depression (10.1%) were also commonly reported. Other notable comorbidities included cardiovascular disease (11.0%), sleep disorders (8.9%), and anxiety (4.7%). Less frequently reported conditions included thyroid disorders (2.5%), dermatological conditions (4.9%), headache/migraine (7.2%), gastrointestinal disorders (0.8%), and other psychological disorders (1.5%).

**Figure 2:**
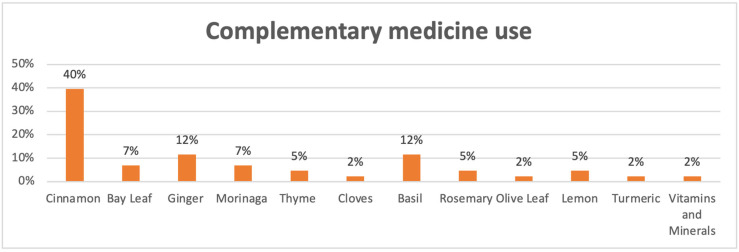
Percentage of use for various complementary medicines.

Figure 2 shows the distribution of complementary medicine use among the participants. Cinnamon was the most used complementary product (40%), followed by basil and ginger (both 12%). Other frequently reported remedies included bay leaf and moringa (7% each), thyme and lemon (5% each), and rosemary (5%). Less commonly used items included cloves, olive leaf, turmeric, and vitamin/mineral supplements (each at 2%).

### 3.2. Unadjusted Associations of the DQoL with Demographic and Socioeconomic Factors, Access to Health Care, Health Behaviors, Diabetes-Related Factors, Comorbidities, and Diabetes Management

The results of the analysis of the bivariate associations between the sample’s characteristics and DQoL are presented in Table 4. For demographic and socioeconomic factors, age, marital status, education level, region of residence, monthly family income, number of family members, and nationality were significantly associated with DQoL scores. Age was significantly associated with QoL, with the highest mean score observed in participants aged 40–50 years (3.43 ± 0.54) and the lowest in those aged 65 or older (3.09 ± 0.58). Marital status showed a strong association, with married individuals reporting the highest QoL (3.43 ± 0.53), while divorced and widowed participants had the lowest scores (3.09 ± 0.55 and 3.12 ± 0.71, respectively). Educational level was another significant factor, with those holding a postgraduate degree having the highest mean QoL (3.70 ± 0.52), while those with less than a high school education reported lower QoL (3.22 ± 0.58). Regional differences were notable, with participants in the central region reporting the highest QoL (3.51 ± 0.58), while those in the eastern region had the lowest (3.14 ± 0.59). Family monthly income has a significant positive association with QoL, with those earning more than 20,000 SR reporting higher scores (3.58 ± 0.68). Additionally, the number of family members also significantly affects QoL, with individuals living with more family members generally reporting higher QoL scores (3.47 ± 0.61). Factors related to access to healthcare that had significant bivariate associations with the DQoL were medical insurance status, settings of diabetes healthcare, diabetes healthcare services, and the number of hospitalizations due to diabetes or other comorbidities. Individuals with medical insurance reported higher QoL scores (3.43 ± 0.60) than non-insured individuals. Additionally, healthcare settings, such as private hospitals (3.56 ± 0.60), were associated with better QoL than other settings. For the most used healthcare services, patients who reported using pharmacy services for diabetes care had higher DQoL scores (3.51 ± 0.58) than those who reported using other services, such as outpatient clinic services (3.38 ± 0.58) or emergency services (2.93 ± 0.53). Moreover, the number of hospitalizations due to diabetes or its complications significantly affects DQoL, with individuals hospitalized fewer times reporting higher scores (*p* < 0.001). Patients with no hospitalizations due to diabetes or its complications had the highest DQoL score (3.44 ± 0.57), while those hospitalized multiple times reported lower scores (3.15 ± 0.67). Similarly, the number of hospitalizations for other health conditions also had a significant impact, with fewer hospitalizations associated with higher QoL (*p* < 0.001). Patients with no hospitalizations due to other health conditions had the highest DQoL score (3.42 ± 0.58), while those hospitalized multiple times reported lower scores (3.15 ± 0.47).

Health behavior and related factors, such as weight status and physical activities, were also linked to QoL. Patients who reported normal weight status had the highest DQoL score (3.43 ± 0.66), while those who reported being underweight had the lowest score (3.17 ± 0.68). Participants exercising ≥ 5 times per week reported the highest scores (3.94 ± 0.50), while those not exercising had lower QoL (3.26 ± 0.59).

Additionally, the presence of diabetes complications, such as neuropathy, nephropathy, diabetic foot, and diabetic coma, was associated with significantly lower QoL, with those experiencing these complications reporting the lowest scores (ranging from 2.88 ± 0.58 to 3.01 ± 0.44).

Comorbidities were associated with significant variations in DQoL. Individuals with cardiovascular disease reported a lower DQoL score (3.19 ± 0.59) than those without cardiovascular disease (3.36 ± 0.58). Similarly, people with hypertension (3.22 ± 0.60) and high triglycerides (3.12 ± 0.60) showed slightly lower DQoL scores compared to those without these conditions (3.39 ± 0.58 and 3.38 ± 0.588, respectively). Notably, individuals with respiratory conditions (3.07 ± 0.62) and sleep disorders (3.02 ± 0.65) experienced the most significant declines in DQoL compared to patients without respiratory conditions (3.39 ± 0.58) and sleep disorders (3.37 ± 0.58). For psychological conditions, only individuals who reported having anxiety (3.01 ± 0.54) and/or depression (2.81 ± 0.44) had the lowest scores as compared to those without these conditions (3.38 ± 0.59 and 3.37 ± 0.59, respectively).

Finally, using other antidiabetic medications (3.26 ± 0.58 vs. 3.40 ± 0.60) and insulin injections (3.28 ± 0.61 vs. 3.41 ± 0.56) were associated with lower QoL than non-users.

### 3.3. Adjusted Associations of the DQoL with Demographic and Socioeconomic Factors, Access to Health Care, Health Behaviors, Diabetes-Related Factors, Comorbidities, and Diabetes Management

Table 5 demonstrates the findings of multivariate association analysis between the sample’s characteristics and DQoL. Demographic and socioeconomic factors, including educational level and region of residence, remained significantly associated with DQoL. In contrast, other demographic and socioeconomic actors were not significantly associated with DQoL in the adjusted analyses. Individuals with less than a high school education had a lower QoL (beta = −1.09; 95% CI = −1.99 to −0.18; *p*-value = 0.018) than those with postgraduate education. Individuals with diabetes from the eastern region reported a worse QoL (beta = −0.97; 95% CI = −1.60 to −0.34; *p*-value = 0.003) compared to those from the southern region, while residents of other regions did not show any significant differences in their DQoL.

Considering access to healthcare, only variations related to the most accessible settings for diabetes healthcare were significantly associated with scores of DQoL. Factors, such as medical insurance status, most frequently used healthcare services, out-of-pocket expenditures for diabetes, the number of hospitalizations due to diabetes or its complications, and the number of hospitalizations due to other health conditions, were significantly associated with DQoL. Individuals who primarily accessed private hospitals reported a significantly higher QoL (beta = 1.09; 95% CI = 0.41 to 1.77; *p*-value = 0.002) than those who reported using small private healthcare clinics or centers.

For variables related to health behavior, only weight status and physical activities sustained independent associations with DQoL. Patients with normal weight reported better QoL compared to those who were obese (beta = 0.54; 95% CI = 0.06 to 1.03; *p*-value = 0.028). For physical activities, individuals who exercised less than five times per week had significantly lower QoL scores. Specifically, individuals who exercised only one or two times per week had a beta coefficient of −1.65 (95% CI = −2.38 to −0.94; *p*-value < 0.001) compared to those who exercised five or more times per week.

The diabetes-related factors, including the presence of diabetic neuropathy and diabetic foot, were significantly associated with DQoL. In contrast, other diabetes-related complications and diabetes duration do not have significant associations with DQoL. The presence of diabetic neuropathy and diabetic foot was associated with poorer QoL. Specifically, those with diabetic neuropathy had significantly lower QoL scores (β = −1.14; 95% CI = −2.02 to −0.26; *p*-value = 0.011). Similarly, those with diabetic foot also experienced a lower QoL (β = −1.18; 95% CI = −2.01 to −0.35; *p*-value = 0.005).

Among the studied comorbid conditions, depression had a significant impact on DQoL. Individuals diagnosed with depression reported a significantly lower QoL (beta = −2.01; 95% CI = −2.93 to −1.10; *p*-value < 0.001) compared to those without depression. Other comorbidities, such as cardiovascular disease and hypertension, did not show significant associations with QoL.

Finally, after conducting adjusted analyses, diabetes management factors, such as metformin, other oral antidiabetic medications, insulin injections, and complementary medicine, were not significantly associated with QoL.

## 4. Discussion

This study revealed that several factors could influence the QoL of individuals with diabetes. Overall, demographic factors (age, marital status, education level, regional differences, family income, number of family members, and nationality), access to healthcare (medical insurance status, most accessible healthcare settings, most commonly used healthcare services, and number of hospitalizations), health behaviors (weight status, and physical activities), and diabetes-related factors (diabetic neuropathy, diabetic nephropathy, diabetic foot, and diabetic coma), comorbidities (cardiovascular disease, hypertension, high triglycerides, respiratory conditions, sleep disorders, anxiety, and depression), and diabetes management (oral antidiabetic use, and insulin injection use) were significantly associated with the participants’ reported QoL.

Although most of the previously mentioned associations were not robust enough to sustain after multivariate analyses, education level, region of residence, most accessible healthcare settings, weight status, physical activities, diabetic neuropathy, diabetic foot, and depression remained significantly associated with diabetes-related QoL.

Individuals with less than a high school education and those with only a high school education reported a lower DQoL than those with postgraduate education. This is consistent with the previous literature suggesting that education level is an essential factor in enhancing self-management and QoL in patients with diabetes [5,20,21]. Likewise, higher educational attainment may enhance health literacy, potentially contributing to better diabetes outcomes and overall QoL [22,23]. Furthermore, individuals in the eastern region reported a significantly lower QoL than those from the southern region (the reference group). Even after adjusting for the impact of other factors, including demographic and socioeconomic factors, access to health care, health behaviors, diabetes-related factors, comorbidities, and diabetes management, this regional difference in QoL could reflect disparities in cultural differences or other unobserved factors that impact diabetes care and QoL.

For access to healthcare, the type of accessible healthcare settings plays a critical role in determining DQoL, regardless of medical insurance status, most used healthcare services, diabetes out-of-pocket expenditures, and the number of hospitalizations. Individuals primarily using private hospitals had higher QoL scores than those who accessed smaller private centers or clinics. This suggests that the quality of healthcare services in these settings, including comprehensive services, improved healthcare infrastructure, and specialized diabetes care in private hospitals, may enhance patient outcomes rather than relying solely on access to care.

Weight status and physical activity had a significant influence on the scores of DQoL, after controlling for all other factors. Patients with normal weight reported a better QoL than obese individuals. Weight and obesity have a well-established association with DQoL, which may be mediated by body image perception [24]. On the other hand, individuals who exercised less frequently (<5 times per week) reported lower QoL scores. These results further emphasize the role of physical activity in improving DQoL [25,26]. This modifiable health behavior was assessed as part of the capability to adopt a healthy lifestyle among the Saudi population in a previous study, and it was found that some socioeconomic factors and perceived health status influenced it [27]. Thus, many structured programs and initiatives have been launched to promote a healthy lifestyle and more physical activity, such as outdoor walking paths and healthy malls, which are accessible to the entire population [28].

For diabetes-related complications, only diabetic neuropathy and diabetic foot are significantly associated with poorer DQoL, while other complications were not significantly associated with DQoL after controlling for other variables. These findings align with previous research, which showed that neuropathy could have the most tremendous negative consequences on the QoL among patients with diabetes [29,30,31]. Although studies about diabetic foot and its impact on QoL are scarce, they indicated that diabetic ulceration could deteriorate the QoL in patients with diabetes [32]. This deterioration in QoL associated with diabetic neuropathy and diabetic foot could be attributed to the severe pain and functional disability that accompany these complications [33].

Conversely, our study indicated no significant impact of retinopathy on diabetes-related QoL. At the same time, Venkataraman et al. suggested its negative influence on scores of the generic measure of health-related QoL (SF-36 v 2) [29].

Concerning physical and mental conditions, only depression remained significantly associated with DQoL after adjusted analyses. Individuals diagnosed with depression reported a significantly lower QoL compared to those without depression. This result indicated the considerable burden that depression may add to patients with diabetes in terms of their QoL. Therefore, it is essential to integrate psychosocial care into diabetes management, with a need for routine screening and appropriate mental health interventions [34]. Moreover, this study sheds light on addressing depression as part of this care, because the type of depression treatment, either psychotherapy or antidepressants, may have varying impacts on physical and mental health outcomes [35].

Surprisingly, our findings demonstrated no robust associations between diabetes management and DQoL. Although individuals who reported using insulin or other antidiabetic medications generally had lower QoL scores in the bivariate analysis, these associations did not persist in the multivariate analysis. This could be due to the influence of mediating factors such as weight status, diabetes-related complications, and depression. Additionally, adherence, treatment burden, and psychosocial context may further shape these relationships [36].

Importantly, the strong positive association observed between frequent physical activity (≥5 times/week) and DQoL supports a dose–response relationship, underlining the clinical importance of promoting regular physical activity in diabetes management strategies.

## 5. Limitations

The findings of this study should be interpreted considering several limitations. First, the cross-sectional design precludes the establishment of causal relationships between diabetes-related quality of life (DQoL) and the examined factors. Second, diabetes diagnosis was based on self-reported data, which may be subject to recall bias. Third, although the mixed-mode approach (online self-administered questionnaires and phone interviews) improved accessibility and geographic reach, it may have introduced response bias. Lastly, the study did not differentiate between type 1 and type 2 diabetes, as participants were included based on self-reported diagnosis without specifying the type of diabetes, which limited the ability to assess type-specific variations in DQoL.

## 6. Conclusions

In conclusion, these findings highlight the importance of targeted interventions that consider factors, such as education, regional disparities, and healthcare settings (regardless of out-of-pocket expenses), to improve outcomes for individuals with diabetes. The results suggest that promoting regular physical activity and addressing weight management could be key strategies for enhancing the QoL in diabetic patients. Additionally, there is a need for integrated care models that address both the physical and psychological aspects of managing diabetes, including diabetic neuropathy, diabetic foot, and comorbid depression. Also, future prospective studies and randomized trials are needed to evaluate the causal impact of structured physical activity and mental health interventions on quality of life and glycemic control in Saudi patients with diabetes.

## Figures and Tables

**Table 1:** Sociodemographic characteristics of the included patients with diabetes (N = 527).

Characteristics		Frequency	%
**Age (years)**			
	Less than 18	35	6.6
	From 18 to less than 23	70	13.3
	More than 23 to less than 30	43	8.2
	From 30 to less than 40	60	11.14
	From 40 to less than 50	103	19.5
	From 50 to less than 60	100	19.0
	From 60 to less than 65	59	11.2
	≥65	57	10.8
**Gender**			
	Male	227	43.1
	Female	300	56.1
**Marital status**			
	Single	144	27.3
	Married	304	57.7
	Divorced	44	8.3
	Widowed	35	6.6
**Educational level**			
	Less than high school	127	24.1
	High school	174	33
	Bachelor’s degree	196	37.2
	Postgraduate	30	5.7
**Occupation**			
	Student	69	13.1
	Employed	183	34.7
	Housewife	136	25.8
	Unemployed and looking for a job	38	7.2
	Retired	74	14.0
	Business	27	5.1
**Region of residence**			
	Central region	213	40.4
	Eastern region	177	33.6
	Western region	63	12.0
	Northern region	7	1.3
	Southern region	67	12.7
**Urban or rural**			
	Urban	494	93.7
	Rural	33	6.3
**Family monthly income**			
	<3000 SR	47	8.9
	3000–<5000 SR	50	9.5
	5000–<10,000 SR	114	21.6
	10,000–<15,000 SR	106	20.1
	15,000–<20,000 SR	85	16.1
	20,000–<25,000 SR	45	8.5
	25,000–<30,000 SR	31	5.9
	≥30,000 SR	49	9.3
**Number of family members**			
	Living alone	54	10.2
	Only two	31	5.9
	3	55	10.4
	4	70	13.3
	5	90	17.1
	6	77	14.6
	7	51	9.7
	8	37	7.0
	≥9	62	11.8
**Nationality**			
	Saudi	468	88.8
	Non-Saudi	59	11.2

**Table 2:** Healthcare access and healthcare utilization.

Characteristics		Frequency	%
**Medical insurance status**			
	Have medical insurance	177	33.6
	Non-insured and use public hospitals and clinics	285	54.1
	Non-insured and use private hospitals and clinics (out-of-pocket)	65	12.3
**Most accessible setting for diabetes-related healthcare**			
	Public hospitals	280	53.1
	Private hospitals	107	20.3
	Public primary health care centers	90	17.1
	Private healthcare centers/clinics	50	9.5
**Most used healthcare services**			
	Outpatient clinic services	421	79.9
	Emergency services	61	11.6
	Pharmacy services	45	8.5
**Diabetes out-of-pocket expenditures**			
	No out-of-pocket expenditures	269	51.0
	<100 SR	34	6.5
	100–< 250 SR	68	12.9
	250–< 500 SR	49	9.3
	500–< 1000 SR	56	10.6
	≥1000 SR	51	9.7
**Number of hospitalizations due to diabetes or its complications**			
	0	372	70.6
	1	58	11.0
	2	56	10.6
	≥3	41	7.8
**Number of hospitalizations due to other health conditions**			
	0	338	64.1
	1	79	15.0
	2	47	8.9
	≥3	63	12.0

**Table 3:** Health behaviors, diabetes-related factors, and comorbidities.

Characteristics		Frequency	%
**Wight status**			
	Underweight	14	2.7
	Normal weight	145	27.5
	Overweight	168	31.9
	Obese	200	38.0
**Following a healthy diet**			
	No	284	53.9
	Yes	243	46.1
**Exercises/physical activities**			
	No exercises	188	35.7
	1 or 2 times per week	206	39.1
	3 or 4 times per week	99	18.8
	≥5 times per week	34	6.5
**Smoking status**			
	Non-smoker	405	76.9
	Past smoker	46	8.7
	Current smoker	76	14.4
**Diabetes-related factors**			
**Diabetes duration (years)**			
	Less than 5	159	30.2
	From 5 to less than 10	101	19.2
	From 10 to less than 15	110	20.9
	From 15 to less than 20	54	10.2
	≥20	103	19.5
**Family history of diabetes**			
	No family history	139	26.4
	2nd grade relatives	74	14.0
	1st grade relatives	227	43.1
	Both 2nd and 1st grades relative	87	16.5
**Diabetes retinopathy**			
	No	462	87.7
	Yes	65	12.3
**Diabetes neuropathy**			
	No	492	93.4
	Yes	35	6.6
**Diabetes nephropathy**			
	No	467	88.6
	Yes	60	11.4
**Diabetic foot**			
	No	501	95.1
	Yes	26	4.9
**Diabetic coma**			
	No	507	96.2
	Yes	20	3.8
**Comorbidities**			
**Presence of other comorbid conditions**			
	No	214	40.6
	Yes	313	59.4
**Diabetes managements**			
**Metformin use**			
	No	187	35.5
	Yes	340	64.5
**Other antidiabetic medication use**			
	No	318	60.3
	Yes	209	39.7
**Insulin injection use**			
	No	250	47.4
	Yes	277	52.6
**Complementary medicine use**			
	No	474	89.9
	Yes	53	10.1

**Table 4:** Bivariate associations of sample characteristics with diabetes-related quality of life.

Characteristics		Mean	SD	*p*-Value
**Total**		3.34	0.59	
**Demographic and socioeconomic factors**				
**Age (years)**				0.010 ^a^
	Less than 18	3.34	0.74	
	From 18 to less than 23	3.32	0.66	
	More than 23 to less than 30	3.19	0.54	
	From 30 to less than 40	3.35	0.56	
	From 40 to less than 50	3.43	0.54	
	From 50 to less than 60	3.42	0.59	
	From 60 to less than 65	3.43	0.53	
	≥65	3.09	0.58	
**Gender**				0.107 ^b^
	Male	3.37	0.63	
	Female	3.33	0.56	
**Marital status**				<0.001 ^a^
	Single	3.28	0.66	
	Married	3.43	0.53	
	Divorced	3.09	0.56	
	Widowed	3.12	0.71	
**Educational level**				0.001 ^a^
	Less than high school	3.22	0.58	
	High school	3.33	0.66	
	Bachelor’s degree	3.37	0.52	
	Postgraduate	3.70	0.52	
**Occupation**				0.787 ^a^
	Student	3.32	0.68	
	Employed	3.38	0.59	
	Housewife	3.30	0.55	
	Unemployed and looking for a job	3.28	0.571	
	Retired	3.39	0.58	
	Business	3.29	0.69	
**Region of residence**				<0.001 ^a^
	Central region	3.51	0.58	
	Eastern region	3.14	0.59	
	Western region	3.42	0.65	
	Northern region	3.25	0.59	
	Southern region	3.30	0.38	
**Urban or rural**				0.530 ^b^
	Urban	3.35	0.59	
	Rural	3.28	0.57	
**Family monthly income**				0.001 ^a^
	<3000 SR	3.09	0.55	
	3000–< 5000 SR	3.24	0.52	
	5000–< 10,000 SR	3.27	0.50	
	10,000–< 15,000 SR	3.33	0.58	
	15,000–< 20,000 SR	3.38	0.61	
	20,000–< 25,000 SR	3.58	0.68	
	25,000–< 30,000 SR	3.50	0.62	
	≥30,000 SR	3.51	0.68	
**Number of family members**				<0.001 ^a^
	Living alone	3.08	0.58	
	Only two	3.21	0.48	
	3	3.19	1.61	
	4	3.30	0.54	
	5	3.41	0.64	
	6	3.37	0.53	
	7	3.57	0.63	
	8	3.42	0.51	
	≥9	3.47	0.61	
**Nationality**				0.016 ^b^
	Saudi	3.36	0.59	
	Non-Saudi	3.17	0.58	
**Access to healthcare**				
**Medical insurance status**				0.022 ^a^
	Have medical insurance	3.43	0.60	
	Non-insured and use public hospitals and clinics	3.28	0.58	
	Non-insured and use private hospitals and clinics (out-of-pocket)	3.39	0.61	
**Most accessible setting for diabetes healthcare**				<0.001 ^a^
	Public hospitals	3.36	0.58	
	Private hospitals	3.56	0.61	
	Public primary health care centers	3.11	0.54	
	Private healthcare centers/clinics	3.20	0.55	
**Most used healthcare services**				<0.001 ^a^
	Outpatient clinic services	3.38	0.58	
	Emergency services	2.93	0.53	
	Pharmacy services	3.51	0.58	
**Diabetes out-of-pocket expenditures**				0.324 ^a^
	No out-of-pocket expenditures	3.38	0.60	
	<100 SR	3.50	0.50	
	100–< 250 SR	3.34	0.52	
	250–< 500 SR	3.42	0.69	
	500–< 1000 SR	3.24	0.59	
	≥1000 SR	3.24	0.65	
**Number of hospitalizations due to diabetes or its complications**				<0.001 ^a^
	0	3.44	0.57	
	1	3.13	0.62	
	2	3.06	0.47	
	≥3	3.15	0.67	
**Number of hospitalizations due to other health conditions**				<0.001 ^a^
	0	3.42	0.58	
	1	3.27	0.63	
	2	3.14	0.64	
	≥3	3.15	0.47	
**Health behaviors**				
**Wight status**				0.024 ^a^
	Underweight	3.17	0.68	
	Normal weight	3.43	0.66	
	Overweight	3.39	0.57	
	Obese	3.26	0.54	
**Following a healthy diet**				0.472 ^b^
	No	3.36	0.60	
	Yes	3.32	0.59	
**Exercises/physical activities**				<0.001 ^a^
	No exercises	3.26	0.59	
	1 or 2 times per week	3.29	0.58	
	3 or 4 times per week	3.40	0.55	
	≥5 times per week	3.94	0.50	
**Smoking status**				0.594 ^a^
	Non-smoker	3.35	0.57	
	Past smoker	3.35	0.61	
	Current smoker	3.28	0.69	
**Diabetes-related factors**				
**Diabetes duration (years)**				0.415 ^a^
	Less than 5	3.42	0.53	
	From 5 to less than 10	3.30	0.62	
	From 10 to less than 15	3.33	0.60	
	From 15 to less than 20	3.34	0.69	
	≥20	3.29	0.59	
**Family history of diabetes**				0.350 ^a^
	No family history	3.40	0.61	
	2nd grade relatives	3.37	0.49	
	1st grade relatives	3.33	0.59	
	Both 2nd and 1st grades relative	3.26	0.65	
**Diabetes retinopathy**				0.149 ^b^
	No	3.36	0.59	
	Yes	3.24	0.64	
**Diabetes neuropathy**				<0.001 ^b^
	No	3.37	0.58	
	Yes	2.95	0.55	
**Diabetes nephropathy**				<0.001 ^b^
	No	3.39	0.58	
	Yes	2.94	0.56	
**Diabetic foot**				<0.001 ^b^
	No	3.37	0.58	
	Yes	2.88	0.58	
**Diabetic coma**				0.010 ^b^
	No	3.36	0.59	
	Yes	3.01	0.44	
**Comorbidities**				
**Presence of other comorbid conditions**				0.003 ^b^
	No	3.43	0.58	
	Yes	3.28	0.59	
**Cardiovascular disease**				0.039 ^b^
	No	3.36	0.58	
	Yes	3.19	0.59	
**Hypertension**				0.003 ^b^
	No	3.39	0.58	
	Yes	3.22	0.60	
**High cholesterol**				0.051 ^b^
	No	3.37	0.58	
	Yes	3.25	0.64	
**High triglycerides**				<0.001 ^b^
	No	3.38	0.58	
	Yes	3.12	0.60	
**Headache/migraine**				0.444 ^b^
	No	3.34	0.60	
	Yes	3.41	0.49	
**Respiratory conditions**				<0.001 ^b^
	No	3.39	0.58	
	Yes	3.07	0.62	
**Musculoskeletal disease**				0.145 ^b^
	No	3.36	0.58	
	Yes	3.25	0.66	
**Gastrointestinal disorders**				0.110 ^b^
	No	3.35	0.59	
	Yes	2.85	0.50	
**Sleep disorders**				<0.001 ^b^
	No	3.37	0.58	
	Yes	3.02	0.65	
**Thyroid disorders**				0.669 ^b^
	No	3.34	0.59	
	Yes	3.27	0.63	
**Dermatological conditions**				
	No	3.35	0.60	0.347 ^b^
	Yes	3.24	0.49	
**Anxiety**				<0.001 ^b^
	No	3.38	0.59	
	Yes	3.01	0.54	
**Depression**				<0.001 ^b^
	No	3.37	0.59	
	Yes	2.81	0.44	
**Other psychological conditions**				0.250 ^b^
	No	3.35	0.59	
	Yes	3.10	0.39	
**Diabetes managements**				
**Metformin use**				0.121 ^b^
	No	3.29	0.62	
	Yes	3.37	0.57	
**Other antidiabetic medication use**				0.007 ^b^
	No	3.40	0.60	
	Yes	3.26	0.58	
**Insulin injection use**				0.007 ^b^
	No	3.41	0.56	
	Yes	3.28	0.61	
**Complementary medicine use**				0.999 ^b^
	No	3.34	0.59	
	Yes	3.34	0.60	

Note: ^a^: One-way ANOVA test; ^b^: Independent *t*-test.

**Table 5:** Multivariate analyses of the associations between diabetes-related quality of life and all factors related to patients with diabetes.

Characteristics		Beta	95% CI	*p*-Value
**Demographic and socioeconomic factors**				
**Age (years)**				
	Less than 18	0.61	(−0.83, 2.05)	0.404
	From 18 to less than 23	0.08	(−1.13, 1.28)	0.902
	More than 23 to less than 30	−0.40	(−1.52, 0.72)	0.482
	From 30 to less than 40	0.03	(−0.91, 0.95)	0.956
	From 40 to less than 50	−0.10	(−0.95, 0.76)	0.826
	From 50 to less than 60	0.40	(−0.35, 1.16)	0.297
	From 60 to less than 65	0.36	(−0.38, 1.10)	0.341
	≥65	**Reference group**		
**Gender**				
	Male	0.30	(−0.17, 0.77)	0.212
	Female	**Reference group**		
**Marital status**				
	Single	−0.64	(−1.78, 0.51)	0.277
	Married	−0.18	(−1.02, 0.66)	0.680
	Divorced	−0.19	(−1.13, 0.74)	0.685
	Widowed	**Reference group**		
**Educational level**				
	Less than high school	−1.09	(−1.99, −0.18)	0.018
	High school	−0.83	(−1.63, −0.03)	0.043
	Bachelor’s degree	−0.65	(−1.40, 0.10)	0.090
	Postgraduate	**Reference group**		
**Occupation**				
	Student	−0.11	(−1.22, 1.00)	0.851
	Employed	0.23	(−0.59, 1.04)	0.586
	Housewife	0.63	(−0.28, 1.54)	0.175
	Unemployed and looking for a job	0.54	(−0.52, 1.61)	0.316
	Retired	0.29	(−0.60, 1.17)	0.529
	Business	**Reference group**		
**Region of residence**				
	Central region	0.00	(−0.61, 0.63)	0.983
	Eastern region	−0.97	(−1.60, −0.34)	0.003
	Western region	0.34	(−0.36, 1.04)	0.341
	Northern region	−0.48	(−2.06, 1.10)	0.553
	Southern region	**Reference group**		
**Urban or rural**				
	Urban	0.08	(−0.63, 0.78)	0.835
	Rural	**Reference group**		
**Family monthly income**				
	<3000 SR	−0.72	(−1.56, 0.11)	0.089
	3000–< 5000 SR	−0.42	(−1.21, 0.36)	0.290
	5000–< 10,000 SR	−0.65	(−1.32, 0.03)	0.059
	10,000–< 15,000 SR	−0.60	(−1.27, 0.07)	0.079
	15,000–< 20,000 SR	−0.53	(−1.21, 0.15)	0.129
	20,000–< 25,000 SR	0.40	(−0.37, 1.16)	0.311
	25,000–< 30,000 SR	−0.37	(−1.24, 0.50)	0.404
	≥30,000 SR	**Reference group**		
**Number of family members**				
	Living alone	−0.74	(−1.52, 0.05)	0.067
	Only two	0.11	(−0.76, 0.99)	0.802
	3	−0.60	(−1.36, 0.16)	0.122
	4	−0.38	(−1.07, 0.31)	0.280
	5	0.31	(−0.33, 0.94)	0.341
	6	−0.21	(−0.86, 0.43)	0.518
	7	0.53	(−0.18, 1.23)	0.141
	8	0.01	(−0.75, 0.78)	0.968
	≥9	**Reference group**		
**Nationality**				
	Saudi	0.56	(−0.03, 1.16)	0.065
	Non-Saudi	**Reference group**		
**Access to healthcare**				
**Medical insurance status**				
	Have medical insurance	−0.08	(−0.66, 0.50)	0.797
	Non-insured and use public hospitals and clinics	−0.26	(−0.90, 0.38)	0.430
	Non-insured and use private hospitals and clinics (out-of-pocket)	**Reference group**		
**Most accessible setting for diabetes healthcare**				
	Public hospitals	0.62	(−0.08, 1.31)	0.083
	Private hospitals	1.09	(0.41, 1.77)	0.002
	Public primary health care centers	−0.12	(−0.92, 0.68)	0.763
	Private healthcare centers/clinics	**Reference group**		
**Most used healthcare services**				
	Outpatient clinic services	0.01	(−0.60, 0.61)	0.985
	Emergency services	−0.51	(−1.32, 0.30)	0.213
	Pharmacy services	**Reference group**		
**Diabetes out-of-pocket expenditures**				
	No out-of-pocket expenditures	0.55	(−0.08, 1.17)	0.09
	<100 SR	0.17	(−0.72, 1.06)	0.71
	100–<250 SR	0.20	(−0.54, 0.91)	0.61
	250–<500 SR	0.43	(−0.33, 1.19)	0.262
	500–<1000 SR	−0.40	(−1.15, 0.35)	0.29
	≥1000 SR	**Reference group**		
**Number of hospitalizations due to diabetes or its complications**				
	0	0.16	(−0.55, 0.86)	0.665
	1	0.01	(−0.80, 0.82)	0.983
	2	−0.16	(−0.94, 0.62)	0.684
	≥3	**Reference group**		
**Number of hospitalizations due to other health conditions**				
	0	0.16	(−0.20, 1.01)	0.186
	1	0.01	(−0.37, 0.99)	0.375
	2	−0.16	(−0.62, 0.86)	0.753
	≥3	**Reference group**		
**Health behaviors**				
**Wight status**				
	Underweight	−0.33	(−1.45, 0.79)	0.567
	Normal weight	0.54	(0.06, 1.03)	0.028
	Overweight	−0.01	(−0.43, 0.41)	0.953
	Obese	**Reference group**		
**Following a healthy diet**				
	No	0.04	(−0.33, 040)	0.84
	Yes	**Reference group**		
**Exercises/physical activities**				
	No exercises	−1.83	(−2.56, −1.10)	<0.001
	1 or 2 times per week	−1.65	(−2.38, −0.94)	<0.001
	3 or 4 times per week	−1.66	(−2.43, −0.88)	<0.001
	≥5 times per week	**Reference group**		
**Smoking status**				
	Non-smoker	0.22	(−0.34, 0.78)	0.435
	Past smoker	−0.11	(−0.83, 0.60)	0.756
	Current smoker	**Reference group**		
**Diabetes-related factors**				
**Diabetes duration (years)**				
	Less than 5	0.09	(−0.51, 0.70)	0.765
	From 5 to less than 10	−0.37	(−0.98, 0.25)	0.243
	From 10 to less than 15	−0.42	(−0.98, 0.14)	0.144
	From 15 to less than 20	−0.10	(−0.78, 0.58)	0.772
	≥20	**Reference group**		
**Family history of diabetes**				
	No family history	−0.22	(−0.78, 0.33)	0.433
	2nd grade relatives	−0.33	(−0.95, 0.30)	0.303
	1st grade relatives	−0.16	(−0.65, 0.34)	0.534
	Both 2nd and 1st grades relative	**Reference group**		
**Diabetes retinopathy**				
	No	**Reference group**		
	Yes	0.51	(−0.03, 1.05)	0.066
**Diabetes neuropathy**				
	No	**Reference group**		
	Yes	−1.14	(−2.02, −0.26)	0.011
**Diabetes nephropathy**				
	No	**Reference group**		
	Yes	−0.45	(−1.14, 0.25)	0.208
**Diabetic foot**				
	No	**Reference group**		
	Yes	−1.18	(−2.01 −0.35)	0.005
**Diabetic coma**				
	No	**Reference group**		
	Yes	−0.68	(−1.61, 0.25)	0.153
**Comorbidities**				
**Cardiovascular disease**				
	No	**Reference group**		
	Yes	0.17	(−0.44, 0.79)	0.581
**Hypertension**				
	No	**Reference group**		
	Yes	0.06	(−0.40, 0.53)	0.795
**High cholesterol**				
	No	**Reference group**		
	Yes	−0.06	(−0.55, 0.43)	0.807
**High triglycerides**				
	No	**Reference group**		
	Yes	−0.37	(−0.97, 0.230)	0.227
**Headache/migraine**				
	No	**Reference group**		
	Yes	0.52	(−0.15, 1.19)	0.130
**Respiratory conditions**				
	No	**Reference group**		
	Yes	−0.12	(−0.904, 0.66)	0.764
**Musculoskeletal disease**				
	No	**Reference group**		
	Yes	0.19	(−0.33, 0.70)	0.470
**Gastrointestinal disorders**				
	No	**Reference group**		
	Yes	−0.87	(−2.84, 1.10)	0.386
**Sleep disorders**				
	No	**Reference group**		
	Yes	−0.37	(−1.32, 0.59)	0.452
**Thyroid disorders**				
	No	**Reference group**		
	Yes	−0.03	(−1.07, 1.00)	0.948
**Dermatological conditions**				
	No	**Reference group**		
	Yes	0.53	(−0.30, 1.37)	0.211
**Anxiety**				
	No	**Reference group**		
	Yes	−0.31	(−0.98, 0.37)	0.369
**Depression**				
	No	**Reference group**		
	Yes	−2.01	(−2.93, − 1.10)	<0.001
**Other psychological conditions**				
	No	**Reference group**		
	Yes	1.05	(−0.43, 2.53)	0.164
**Diabetes managements**				
**Metformin use**				
	No	**Reference group**		
	Yes	0.33	(−0.07, 0.72)	0.108
**Other antidiabetic medication use**				
	No	**Reference group**		
	Yes	−0.37	(−0.75, 0.00)	0.052
**Insulin injection use**				
	No	**Reference group**		
	Yes	−0.01	(−0.45, 0.42)	0.958
**Complementary medicine use**				
	No	**Reference group**		
	Yes	−0.35	(−0.91, 0.21)	0.215

## Data Availability

The submitted manuscript included all essential data. The corresponding author can request the data utilized in or analyzed in this study.

## Supplementary Materials

The following supporting information can be downloaded at: https://www.mdpi.com/article/10.3390/medicina61091583/s1, Table S1: STROBE checklist.
