# Supplementary material for: Determinants of Diabetes-Related Quality of Life in Saudi Arabia: A Nationwide Survey-Based Assessment of Demographic, Health, and Complication-Related Influences
Source: Medicina (Kaunas). 2025 Sep 1;61(9):1583. doi: 10.3390/medicina61091583 (PMC12471931; doi:10.3390/medicina61091583)
Supplement: Supplementary file 1 [file medicina-61-01583-s001.zip › medicina-3771935-supplementary.pdf]

STROBE Statement—Checklist of items that should be included in reports of ***cross-sectional studies***

	Item No	Recommendation	Page No.	Relevant text from manuscript
**Title and abstract**	1	(*a*) Indicate the study's design with a commonly used term in the title or the abstract	1	Title
		(*b*) Provide in the abstract an informative and balanced summary of what was done and what was found	1	Abstract section
**Introduction**				
Background/rationale	2	Explain the scientific background and rationale for the investigation being reported	2	Second and third paragraphs of the introduction
Objectives	3	State specific objectives, including any prespecified hypotheses	2,3	Last paragraph of the introduction
**Methods**				
Study design	4	Present key elements of study design early in the paper	3	2.1. Study Design
Setting	5	Describe the setting, locations, and relevant dates, including periods of recruitment, exposure, follow-up, and data collection	3	2.3. Procedures
Participants	6	(*a*) Give the eligibility criteria, and the sources and methods of selection of participants	3	2.3. Procedures
Variables	7	Clearly define all outcomes, exposures, predictors, potential confounders, and effect modifiers. Give diagnostic criteria, if applicable	3,4	2.4. Measures
Data sources/ measurement	8*	For each variable of interest, give sources of data and details of methods of assessment (measurement). Describe comparability of assessment methods if there is more than one group	*3*	2.4. Measures
Bias	9	Describe any efforts to address potential sources of bias	3	2.4. Measures
Study size	10	Explain how the study size was arrived at	3	2.2. Sample Size
Quantitative variables	11	Explain how quantitative variables were handled in the analyses. If applicable, describe which groupings were chosen and why	4	2.5. Statistical Analysis
Statistical methods	12	(*a*) Describe all statistical methods, including those used to control for confounding	4	2.5. Statistical Analysis

		(*b*) Describe any methods used to examine subgroups and interactions	4	2.5. Statistical Analysis
		(*c*) Explain how missing data were addressed	-	Not encountered
		(*d*) If applicable, describe analytical methods taking account of sampling strategy	-	-
		(*e*) Describe any sensitivity analyses	4	2.5. Statistical Analysis
**Results**				
Participants	13*	(a) Report numbers of individuals at each stage of study—eg numbers potentially eligible, examined for eligibility, confirmed eligible, included in the study, completing follow-up, and analysed	4	First paragraph
		(b) Give reasons for non-participation at each stage	4	First paragraph
		(c) Consider use of a flow diagram	-	-
Descriptive data	14*	(a) Give characteristics of study participants (eg demographic, clinical, social) and information on exposures and potential confounders		3.1. Sample's Characteristics
		(b) Indicate number of participants with missing data for each variable of interest	-	Not encountered
Outcome data	15*	Report numbers of outcome events or summary measures	10	3.2. Unadjusted Associations of the DQoL with Demographic and Socioeconomic Factors, Access to Health Care, Health Behaviors, Diabetes-Related Factors, Comorbidities, and Diabetes Management
Main results	16	(*a*) Give unadjusted estimates and, if applicable, confounder-adjusted estimates and their precision (eg, 95% confidence interval). Make clear which confounders were adjusted for and why they were included	10-22	Table 4 and Table 5
		(*b*) Report category boundaries when continuous variables were categorized	5-9	Table 1, Table 2 and Table 3
		(*c*) If relevant, consider translating estimates of relative risk into absolute risk for a meaningful time period	-	-
Other analyses	17	Report other analyses done—eg analyses of subgroups and	-	-

		interactions, and sensitivity analyses		
**Discussion**				
Key results	18	Summarise key results with reference to study objectives		
Limitations	19	Discuss limitations of the study, taking into account sources of potential bias or imprecision. Discuss both direction and magnitude of any potential bias	23-24	Limitations section
Interpretation	20	Give a cautious overall interpretation of results considering objectives, limitations, multiplicity of analyses, results from similar studies, and other relevant evidence	22-23	Line 343 to 420
Generalisability	21	Discuss the generalisability (external validity) of the study results	22	First and second paragraphs
**Other information**				
Funding	22	Give the source of funding and the role of the funders for the present study and, if applicable, for the original study on which the present article is based	24	Funding statement

*Give information separately for exposed and unexposed groups.

Note: An Explanation and Elaboration article discusses each checklist item and gives methodological background and published examples of transparent reporting. The STROBE checklist is best used in conjunction with this article (freely available on the Web sites of PLoS Medicine at http://www.plosmedicine.org/, Annals of Internal Medicine at http://www.annals.org/, and Epidemiology at http://www.epidem.com/). Information on the STROBE Initiative is available at www.strobe-statement.org.
